# Dietary Suberic Acid Protects Against UVB-Induced Skin Photoaging in Hairless Mice

**DOI:** 10.3390/nu11122948

**Published:** 2019-12-04

**Authors:** Wesuk Kang, Dabin Choi, Taesun Park

**Affiliations:** Department of Food and Nutrition, Brain Korea 21 PLUS Project, Yonsei University, 50 Yonsei-ro, Seodaemun-gu, Seoul 120-749, Korea

**Keywords:** suberic acid, photoaging, UV, collagen, hyaluronic acid

## Abstract

Ultraviolet (UV) radiation is a major cause of skin photoaging, which is mainly characterized by dryness and wrinkle formation. In the current study, we investigated the anti-photoaging effects of dietary suberic acid, a naturally occurring photochemical, using UVB-irradiated hairless mice. Mice were exposed to UVB three times weekly and fed diets containing three different suberic acid concentrations (0.05%, 0.1% and 0.2%) for 10 weeks. It was found that suberic acid inhibited UVB-induced skin dryness, wrinkle formation, and epidermal thickness in hairless mice. In parallel with phenotypic changes, suberic acid attenuated UVB-induced matrix metalloproteinase (*MMP*) genes (*MMP1a*, *MMP1b, MMP3*, and *MMP9*), while accelerating collagen genes including collagen type I alpha 1 chain (*COL1A1*), *COL1A2*, and *COL3A1* and hyaluronic acid synthases genes (*HAS1*, *HAS2* and *HAS3*). We further demonstrated that suberic acid upregulated the molecules involved in the transforming growth factor–β (TGF-β)/SMAD pathway, but downregulated the molecules participating in the mitogen-activated protein kinase (MAPK)/activator protein 1 (AP-1) signaling in UVB-irritated hairless mice. Collectively, we propose that suberic acid may be a promising agent for treating skin photoaging.

## 1. Introduction

Cutaneous aging is caused by intrinsic and extrinsic factors. Intrinsic aging is an inevitable process that causes fine wrinkles, thin skin, and gradual dermal atrophy. On the other hand, extrinsic aging is induced by environmental risks including sun exposure and poor nutrition, leading to dry skin, coarse wrinkles, a rough-textured appearance, and elasticity loss [1,2]. Particularly, chronic solar ultraviolet (UV) exposure, especially UVB, is the major environmental cause of cutaneous aging and thus, extrinsic aging is also called photoaging [3]. It is well documented that chronic and repetitive UVB exposure results in extracellular matrix (ECM) and dermal connective tissue damages. The hallmarks of this UVB-induced ECM remodeling include enzymatic degradation and reduced collagen and hyaluronic acid synthesis, causing skin dryness and wrinkling [4,5].

Among the mediators influencing ECM composition in skin tissues during the photoaging process, transforming growth factor-beta (TGF-β) is a major modulator of ECM synthesis, as it regulates the levels of ECM network components including collagens, fibronectin, and hyaluronic acid [6,7,8,9]. Indeed, TGF-β receptor knockout results in reduced collagen content and remodeling in mouse skin [10]. On the other hand, it is well known that mitogen-activated protein kinase (MAPK) signaling plays an important role in ECM constituent degradation [11,12]. MAPK activation stimulates activator protein 1 (AP-1) transcription factors (e.g., c-Jun and c-Fos). UVB-activated AP-1 then induces matrix metalloproteinase (*MMP*) gene transcription in both keratinocytes and fibroblasts. Owing to MMP activation, collagen and other components (e.g., hyaluronic acid) are degraded in the dermal ECM [1,13].

Since reduction of procollagen in the dermal fibroblast is a well-known phenotype for photoaging, we performed a cell-based assay using HS68 cells to screen chemical libraries for small molecules that could increase procollagen synthesis. Through this process, we found that suberic acid treatment resulted in a marked increase in procollagen content. Suberic acid is a colorless crystalline dibasic acid, with formula C_8_O_4_H_14_ and is abundant in castor, *Hibiscus syriacus*, and *Vernonia galamensis* [14,15]. In the present study, we investigated whether suberic acid inhibited UVB-induced skin photoaging including dryness and wrinkles in hairless mouse skin, and if so, whether this effect was related to TGF-β and MAPK pathway modulation.

## 2. Materials and Methods

### 2.1. Materials

Human dermal fibroblast cell line (HS68) was purchased from American Type Culture Collection (Manassas, VA, USA). Dulbecco’s modified Eagle medium (DMEM) and fetal bovine serum (FBS) were obtained from HyClone (Logan, UT, USA) and penicillin-streptomycin was sourced from Gibco (Grand Island, NE, USA). Suberic acid, Avertin, 3-(4,5-dimethylthiazol-2-yl)-2,5-diphenyltetrazolium bromide (MTT), dimethyl sulfoxide (DMSO), and 10% formalin solution were purchased from Sigma-Aldrich (St. Louis, MO, USA). The antibodies against, protein kinase A catalytic subunit (PKA Cα; 1:1000), TGF-β (1:1000), SMAD 2/3 (1:1000), p-SMAD 2/3 (1:1000), p38 (1:1000), p-p38 (1:1000), c-Jun N-terminal kinase (JNK; 1:1000), p-JNK (1:1000), extracellular-signal-regulated kinase (ERK; 1:1000), p-ERK (1:1000), c-Jun (1:1000), p-c-Jun (1:1000), c-Fos (1:1000) p-c-Fos (1:1000), and glyceraldehyde 3-phosphate dehydrogenase (GAPDH; 1:5000) were purchased from Cell Signaling (Danvers, MA, USA). The horseradish peroxidase-conjugated anti-rabbit IgG antibody (1:10,000) was sourced from Santa Cruz Biotechnology Inc. (Santa Cruz, CA, USA). Trizol and SuperScript reverse transcriptase were purchased from Invitrogen (Carlsbad, CA, USA). Bradford reagent, electrochemiluminescence (ECL) detection reagent, and iQ SYBR green supermix were purchased from BioRad (Hercules, CA, USA). Bovine serum albumin (BSA) was purchased from LPS solution (Daejeon, Republic of Korea).

### 2.2. Cell Culture

HS68 dermal fibroblasts were incubated in high-glucose DMEM supplemented with 10% FBS and 1% 100 U/mL penicillin-streptomycin in a 5% CO_2_ humidified atmosphere incubator (Sanyo, Osaka, Japan) at 37 °C. The medium was changed every 2–3 days and the cells were passaged at 80% confluency.

### 2.3. Cell Viability Assay

Cell viability was estimated by MTT assay. HS68 cells (2 × 10^4^ cells/well) were transferred into 96-well plates and cultured at 37 °C for 24 h. The cells were then treated with or without 1–400 μM suberic acid and cultured for a further 24 h. The cultured cells were rinsed with phosphate buffered saline (PBS) and exposed to UVB (20 mJ/cm^2^) using a CL-1000M UV crosslinker (UVP, Upland, CA, USA). The cells were exposed to UVB for 8 s at a distance of 22 cm from the light source. The cells were then incubated with the same suberic acid concentration for 24 h in serum-free medium. Subsequently, 4 mg/mL MTT solution was transferred to each well and the cells were cultured for a further 4 h. The supernatant was aspirated and the purple formazan crystals were dissolved in DMSO. Relative absorbance was estimated at 570 nm with an Infinite M200 pro microplate reader (Tecan, Männedorf, Switzerland).

### 2.4. Procollagen I C-terminal Peptide Determination

The HS68 cells (5 × 10^4^ cells/well) were transferred into 24-well plates, pretreated with 12.5, 25, 50, and 100 μM suberic acid, and incubated for 24 h. The cells were then rinsed with PBS and irradiated with UVB as described previously. The UVB-exposed HS68 cells were cultured with serum-free medium containing the same suberic acid concentration (12.5, 25, 50, and 100 μM). The supernatants were harvested after 24 h, and procollagen I C-terminal peptide contents were evaluated in the supernatants with an enzyme-linked immunosorbent assay (ELISA) kit (MK101; Takara, Shiga, Japan) according to manufacturer guidelines. Relative absorbance was estimated at 595 nm with a microplate reader.

### 2.5. Animal Experiments

Six-week-old female albino hairless mice (Skh-1; Orient Bio, Seongnam, Korea) were housed (four per cage) in standard cages with wood chip bedding in a room at 22 ± 2 °C, 50 ± 5% relative humidity, and 12:12 h light–dark conditions. The mice were divided into five groups (n = 8 per group): normal group (control diet), UVB control group (control diet and UVB exposure), 0.05% suberic acid group (diet containing 0.05% suberic acid and UVB exposure), 0.1% suberic acid group (diet containing 0.1% suberic acid and UVB exposure), and 0.2% suberic acid group (diet containing 0.2% suberic acid and UVB exposure). Suberic acid at 0.05, 0.1, and 0.2% was incorporated to replace an equivalent amount of corn starch in AIN-93 basal diet (MP Biomedicals, Irvine, CA, USA). UVB irradiation was conducted as described previously [16]. The mice were exposed to UVB three times weekly at a distance of 22 cm from the light source; the UVB doses were increased weekly in increments of 1 minimal erythemal dose (MED; 1 MED = 100 mJ/cm^2^) up to 4 MED (exposure time was 40–160 s) and maintained at 4 MED thereafter. All animals had ad libitum access to diet and water. UVB irradiation was stopped after 10 weeks, and the mice were intraperitoneally anesthetized with Avertin (500 mg/kg body weight) and euthanized by exsanguination via the carotid artery; dorsal skins were dissected, divided into two parts, and treated as follows: (1) fixed with 10% formalin solution for histochemistry, (2) snap-frozen for gene and protein analyses. All protocols were approved by the Institutional Animal Care and Use Committee (Case Number: IACUC-A-201909-955-01) of the Yonsei Laboratory Animal Research Center.

### 2.6. Assessment of Wrinkle Formation

To access the wrinkling severity, hairless mice were anesthetized and their back photographed before sacrifice. On the skin surface, the skin replicas were cast with silpro (Flexico developments LTD., Tokyo, Japan) and wrinkle severity was calculated by Visioline VL 650 (CK Electronics, Cologne, Germany). The markers for skin wrinkle estimation were total wrinkle area, maximum wrinkle depth, mean depth, and mean length.

### 2.7. Determination of Skin Hyaluronic Acid

The harvested tissues were homogenized with a pestle and mortar in lysis buffer containing 100 mmol/L Tris-HCl (pH 7.4), 100 mmol/L orthovanadate, 50 mmol/L sodium pyrophosphate, 50 mmol/L NaF, 50 mmol/L NaCl, 5 mmol/L EDTA, 1 mmol/L phenylmethanesulfonyl fluoride, 1% Triton X-100, 2 μg/mL aprotinin, 1 μg/mL pepstatin A, and 1 μg/mL leupeptin and lysates were centrifuged at 12,000× *g* for 30 min at 4 °C. The supernatants were collected and hyaluronic acid content was measured with hyaluronic acid Quantikine ELISA kit (R&D systems, Abingdon, UK). Optical density was evaluated at 570 nm with a microplate reader.

### 2.8. Transepidermal Water Loss (TEWL) and Skin Hydration Determination

One week before sacrifice, skin TEWL and hydration of hairless mice were estimated with a Cutometer^®^ MPA580 (Courage & Khazaka, Cologne, Germany). The probe tip was developed as a closed chamber containing two hydration sensors on the edge and a water loss sensor on the top of the chamber. The TEWL and skin hydration values were measured in g/m^2^/h and arbitrary units (AU), respectively.

### 2.9. Histological Analysis

After formalin-fixed skin samples were embedded in paraffin, 7-μm sections were stained with hematoxylin and eosin (H&E) or Masson’s trichrome (MT). Images of the tissues were captured for microscopic evaluation with an Olympus IX71 microscope using an DP-70 controller (Olympus, Center Valley, PA, USA). The epidermal thickness of H&E-stained samples was estimated with ImageJ (National Institute of Health, Bethesda, MD, USA). The collagen density on MT-stained tissues was estimated using Quantity One software (BioRad). Each slide was measured at five spots and the average was calculated.

### 2.10. Cyclic Adenosine Monophosphate (cAMP) Assay

The frozen skin tissue was homogenized with a mortar and pestle and lysed with 0.1 M HCl for 30 min. cAMP concentrations of the collected lysates were then determined using cAMP ELISA kit (Enzo Life Sciences, Farmingdale, NY, USA) following the manufacturer guidelines. In brief, the lysates were neutralized and cAMP conjugate was transferred to the binding sites on an IgG-coated microplate for competition with cAMP. Then, unbound cAMP was removed by washing thrice with PBS. The substrate was then carefully added to the each well to estimate the bound enzyme activity. After the reaction was stopped, the relative optical density was estimated at 450 nm with a microplate reader. The cAMP level was normalized to the total intracellular protein amount: The protein concentration of lysates was determined using the Bradford reagent. BSA was used as the standard.

### 2.11. Western Blotting

The frozen skin tissue was homogenized with a mortar and pestle in 1 mL of the previously mentioned lysis buffer. The lysates were then collected, vortexed, and centrifuged at 12,000× *g* for 10 min at 4 °C. The protein concentrations were evaluated by Bradford assay as described above. The prepared samples were separated in SDS-PAGE gel and transferred onto nitrocellulose membranes (Whatman, Dassel, Germany). After transfer, the membranes were blocked with 5% BSA in TBST [20 mM Tris–HCl buffer, pH 7.6 containing 0.05% Tween-20 and 137 mM NaCl]. The membranes were incubated with primary antibodies overnight at 4 °C, followed by the corresponding secondary antibodies for 1 h at 20 °C. ECL detection reagent was used as a substrate, and the images were captured with a WSE-6100 LuminoGraph (ATTO, Tokyo, Japan). Band intensities were calculated with the Quantity One software, and normalized to those of the control (GAPDH).

### 2.12. Reverse Transcription and Quantitative Real-Time PCR

The frozen skin tissue was pulverized in liquid nitrogen using a pestle and mortar and RNA was isolated using Trizol. RNA was reverse transcribed to generate cDNA with SuperScript IV reverse transcriptase. The reaction was performed at 52 °C for 1 h. Real-time PCR quantification was conducted with a Real-Time PCR system (CFX96 Touch; BioRad) in a 20 μL volume: 10 μL iQ SYBR green supermix, 20 pmol primers and 50 ng cDNA template. The relative gene level in each sample was estimated by the threshold cycle (Ct) method. Before relative gene levels were determined, primer efficiency was validated with a standard curve; primers with >95% efficiency were used. The Ct data for target genes and housekeeping genes (*GAPDH*) were used to calculate ΔCt: ΔCt = Ct (target gene) − Ct (*GAPDH*). Thereafter, the ΔΔCt was created by using the following equation: ΔΔCt = ΔCt (Treated) − ΔCt (Control). The relative quantity values were then determined using the expression of 2^−ΔΔCt^. Primer sets used for collagen type I alpha 1 chain (*COL1A1*), *COL1A2*, *COL3A1*, hyaluronic acid synthases 1 (*HAS1*), *HAS2*, *HAS3*, *MMP1a*, *MMP1b*, *MMP3*, *MMP9* and *GAPDH* are given in Table 1.

### 2.13. Statistical Analysis

Results are shown as the mean ± standard error of mean (SEM). An unpaired Student’s t-test was used to analyze all data comparisons between two groups. All statistical analyses were performed using SPSS 25.0 statistical software (SPSS Inc., Chicago, IL, USA) with significance set at * *P* < 0.05, ** *P* < 0.01, and *** *P* < 0.001.

## 3. Results

### 3.1. Suberic Acid Alleviates Collagen Loss in UVB-Exposed Hs68 Dermal Fibroblasts

**Figure 1:**
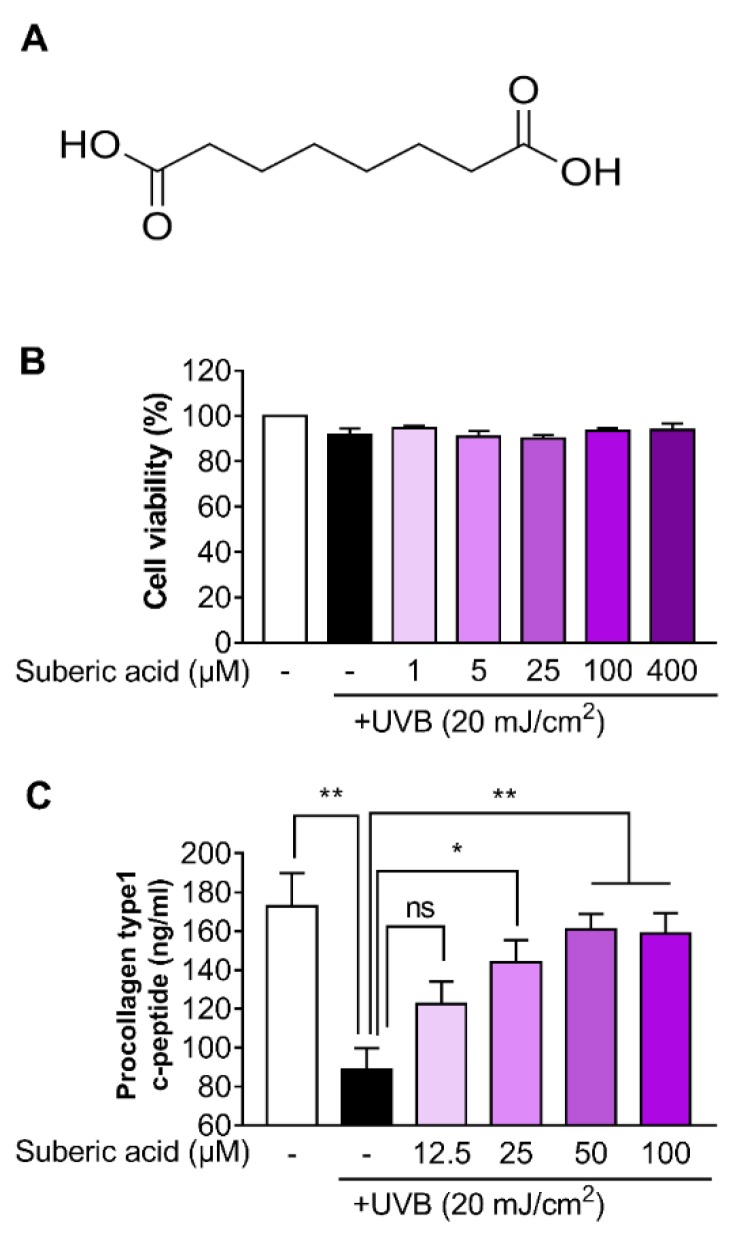
Suberic acid alleviates collagen loss in UVB-exposed Hs68 dermal fibroblasts. (**A**) Structure of suberic acid. (**B**) Viability of Hs68 cells treated with different suberic acid concentrations (1–400 μM) for 24 h after being exposed to 20 mJ/cm^2^ UVB. (**C**) Procollagen type 1 c-peptide concentrations in the supernatant of human Hs68 dermal fibroblasts treated with different suberic acid concentrations (12.5–50 μM) for 24 h after being exposed to 20 mJ/cm^2^ UVB. Results are shown as means ± SEM (*n* = 3). Significant differences between groups are shown as * *P* < 0.05; ** *P* < 0.01; *** *P* < 0.001.

The structure of suberic acid is shown in Figure 1A. We found that suberic acid (up to a concentration of 400 µM) showed no toxicity against UVB-induced Hs68 cells according to MTT assay results (Figure 1B). We also examined whether suberic acid could attenuate collagen loss in UVB-exposed Hs68 cells. Suberic acid significantly reversed procollagen I C-terminal peptide reduction in UVB-exposed Hs68 cell supernatants in a dose-dependent manner, which was saturated around 50 μM (Figure 1C).

### 3.2. Suberic Acid Reduces UVB-Induced Wrinkle Formation in Hairless Mice

**Figure 2:**
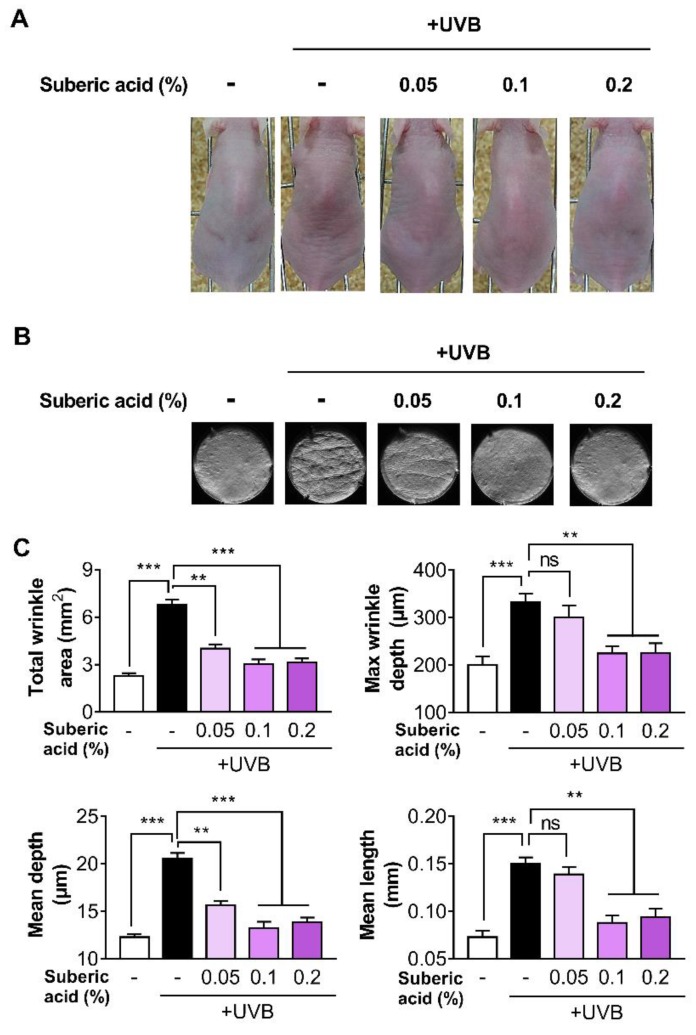
Suberic acid reduces UVB-induced wrinkle formation in hairless mice. The effect of three dietary suberic acid concentrations (0.05%, 0.1% and 0.2%) on (**A**) dorsal skins and (**B**) replicas of hairless mice irritated with UVB for 10 weeks. Histogram of replica analysis at the end of the experiment. (**C**) Total wrinkle area, Maximum wrinkle depth, Mean depth, and Mean length. Results are shown as means ± SEM (*n* = 8). Significant differences between groups are shown as ** *P* < 0.01; *** *P* < 0.001.

To investigate the effect of suberic acid on anti-photoaging, mice were exposed to UVB thrice weekly and fed diets containing three different suberic acid concentrations (0.05%, 0.1%, and 0.2%) for 10 weeks. Daily food intake did not significantly differ among experimental groups (normal group, 4.2 ± 0.12 g/day vs. UVB control group, 4.1 ± 0.09 g/day vs. 0.05% suberic acid group, 4.1±0.09 g/day vs. 0.1% suberic acid group, 4.3 ± 0.15 g/day vs. 0.2% suberic acid group 4.2 ± 0.11 g/day). Supplementation with suberic acid significantly reduced UVB-induced wrinkle formation in a dose-dependent manner (Figure 2A). The analysis of wrinkle severity with wrinkle area, length, and depth in silicon replicas confirmed a significant reduction in wrinkles in mice fed with suberic acid in a concentration-dependent manner (Figure 2B,C).

### 3.3. Suberic Acid Prevents UVB-Induced Skin Dryness in Hairless Mice

**Figure 3:**
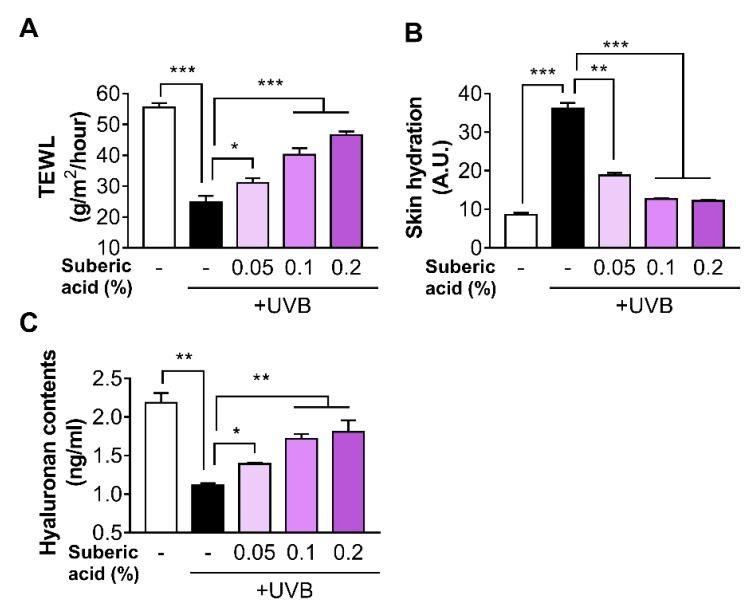
Suberic acid prevents UVB-Induced skin dryness in hairless mice. The effect of three dietary suberic acid concentrations (0.05%, 0.1% and 0.2%) on (**A**) Transepidermal water loss (TEWL) and (**B**) skin hydration on the back of hairless mice exposed to UVB for 10 weeks. (**C**) Changes in skin hyaluronic acid concentration following 10 weeks of continuous dietary suberic acid administration in UVB-exposed hairless mice. Results are shown as means ± SEM (*n* = 8). Significant differences between groups are shown as * *P* < 0.05; ** *P* < 0.01; *** *P* < 0.001.

The suberic acid supplement significantly increased TEWL and decreased skin hydration in a dose-dependent manner (Figure 3A,B). Furthermore, molecular analysis revealed that hyaluronic acid was upregulated in the skin tissues of mice fed with suberic acid in a dose-dependent manner (Figure 3C).

### 3.4. Suberic Acid Suppresses UVB-Induced Epidermal Thickness Increase and Prevents UVB-Induced Collagen Loss in Hairless Mouse Skin

**Figure 4:**
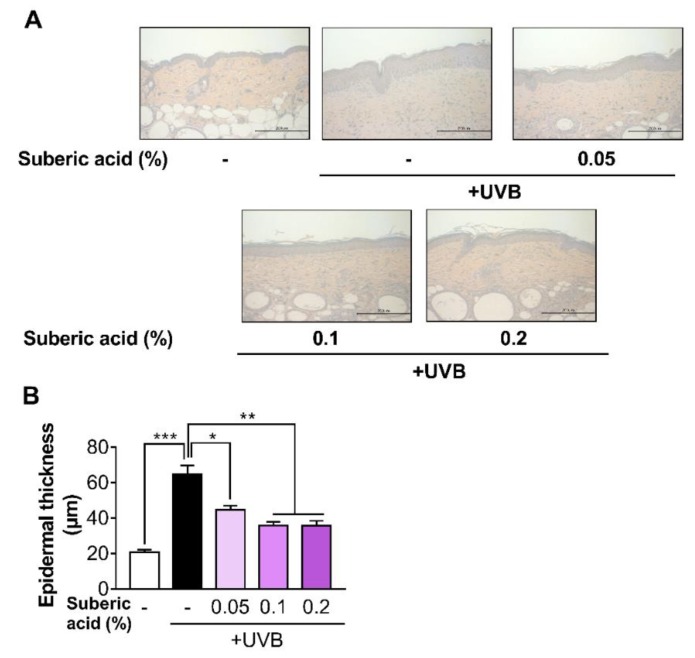
Suberic acid suppresses UVB-induced epidermal thickness increase. (**A**) Representative micrographs of hematoxylin and eosin (H&E) stained skin tissue sections after UVB irradiation for 10 weeks (Scale bar = 200 µm). (**B**) Epidermal thickness of the dorsal skin. Results are shown as means ± SEM (*n* = 8). Significant differences between groups are shown as * *P* < 0.05; ** *P* < 0.01; *** *P* < 0.001.

**Figure 5:**
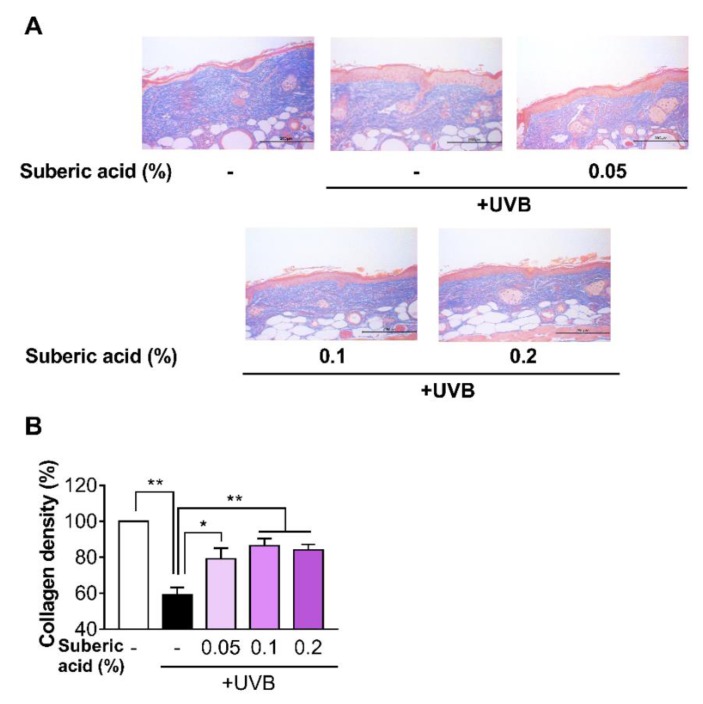
Suberic acid prevents UVB-induced collagen loss in hairless mouse skin. (**A**) Representative micrographs of skin tissue sections stained with Masson’s trichrome stain for collagen after UVB irradiation for 10 weeks (Scale bar = 200 µm). (**B**) Quantitative analysis of dermal collagen density. Results are shown as means ± SEM (*n* = 8). Significant differences between groups are shown as * *P* < 0.05; ** *P* < 0.01.

Increasing dietary suberic acid dose on UVB-exposed hairless mice led to a dose-dependent epidermis thickness decline as confirmed by H&E staining analysis (Figure 4A,B). We then subjected the mouse skin tissues to MT staining to examine the effect of suberic acid on collagen fibers. Suberic acid significantly increased collagen fiber density in the dermis in a dose-dependent manner (Figure 5A,B). As shown in Figure 2, Figure 3, Figure 4 and Figure 5, we used the skin tissues of mice fed diets containing 0.1% suberic acid, mostly representing a saturated effect on anti-photoaging, for all subsequent analysis to explore the possible mechanism of the anti-photoaging effect of suberic acid.

### 3.5. Suberic Acid Upregulates TGF-β Signaling and Downregulates MAPK Pathway Molecules in Hairless Mouse Skin

**Figure 6:**
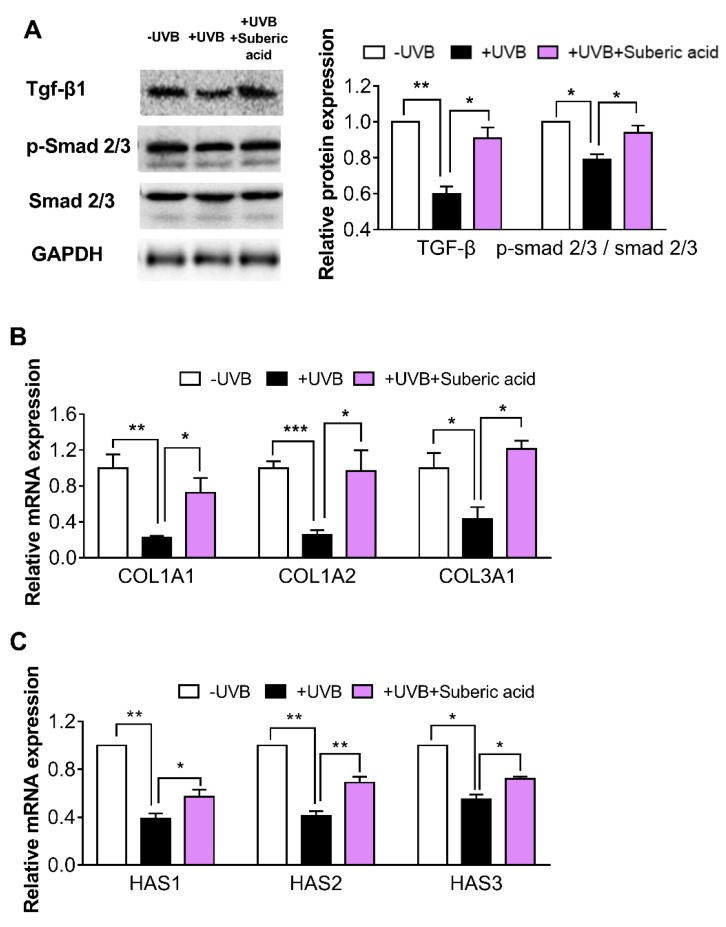
Suberic acid increases the levels of molecules involved in ECM synthesis in hairless mice skin. Effect of dietary suberic acid (0.1%) on (**A**) transforming growth factor-β1 (TGF-β1), p-SMAD, and SMAD 2/3 protein levels and (**B**) collagen type I alpha 1 chain (*COL1A1)*, *COL1A2*, *COL3A1*, (**C**) hyaluronic acid synthase 1 *(HAS1*), *HAS2*, and *HAS3* gene expression in the hairless mouse skin tissue. Results are shown as means ± SEM (*n* = 4–6). Significant differences between groups are shown as * *P* < 0.05; ** *P* < 0.01; *** *P* < 0.001.

**Figure 7:**
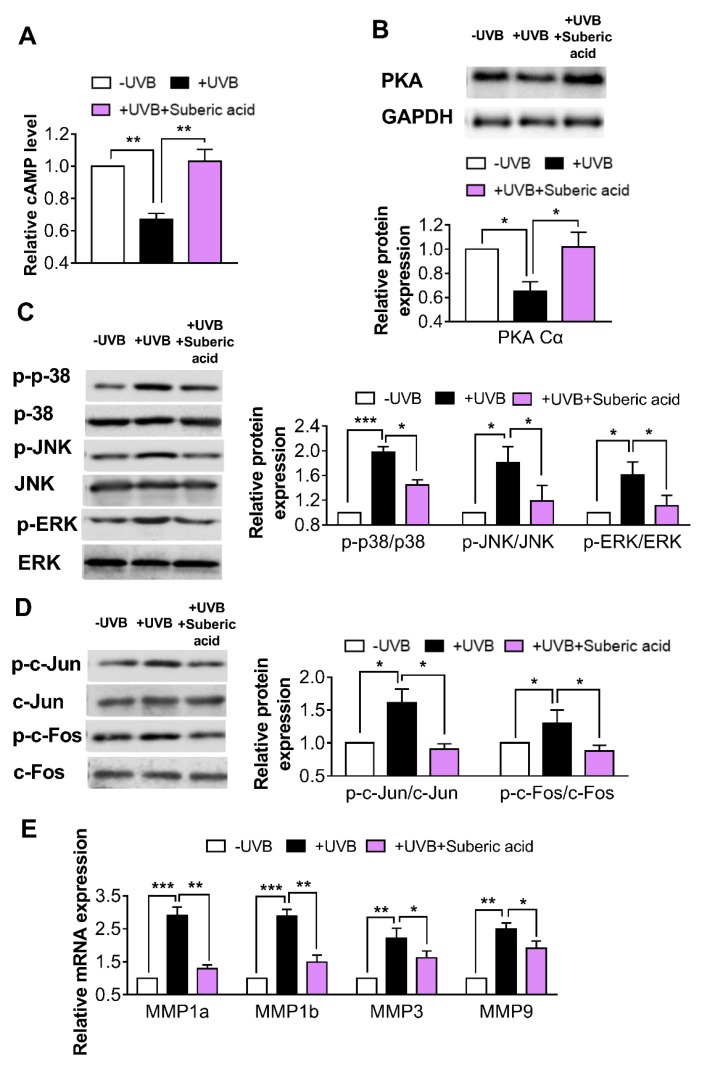
Suberic acid regulates the levels of molecules associated with ECM degradation in hairless mouse skin. Effect of dietary suberic acid (0.1%) on (**A**) cyclic adenosine monophosphate (cAMP) level, (**B**) protein kinase A catalytic subunit (PKA Cα), (**C**) p-p38, p38, p-c-Jun N-terminal kinase (p-JNK), JNK, p-extracellular signal-regulated kinase (p-ERK), ERK, (**D**) p-c-Jun, c-Jun, p-c-Fos, and c-Fos protein levels and (**E**) matrix metalloproteinase 1a *(MMP1a*)*, MMP1b*, *MMP3*, and *MMP9* gene expression in the hairless mouse skin tissue. Results are shown as means ± SEM (*n* = 4–6). Significant difference between groups are shown as * *P* < 0.05; ** *P* < 0.01; *** *P* < 0.001.

In the UVB-irradiated mouse skin tissues, dietary suberic acid (0.1%) significantly increased TGF-β and p-SMAD 2/3 protein expression and *COL1A1*, *COL1A2*, *COL3A1*, *HAS1*, *HAS2*, and *HAS3* gene expression (Figure 6A–C). Suberic acid also strongly increased cAMP concentration and PKA Cα protein expression (Figure 7A,B) and led to MAPK signaling downstream molecule reprogramming in UVB-exposed mouse skin tissues: p-p38, p-ERK, p-JNK, p-c-Fos, and p-c-Jun protein expression and *MMP1a, MMP1b*, *MMP3*, and *MMP9* gene expression were significantly decreased (Figure 7C–E).

## 4. Discussion

In the current study, we used UVB light to cause experimental photoaging in hairless mice. UV light is classified into three zones: UVA (315 to 400 nm), UVB (280 to 315 nm), and UVC (100 to 280 nm). The stratospheric ozone blocks UVC, but UVA and UVB penetrate this layer. While UVA occupies around 95% of UV in terrestrial solar light, UVB accounts for only about 5% UV [17,18]. Although UVA is more abundant than UVB in the sunlight, it has been accepted that UVB is the main factor for UV-induced photoaging owing to its strong energy. The differential result of UVA and UVB irradiation has been reported in the rodent model: While UVA irradiation was only related to minimal dermal effects with normal epidermis, UVB irradiation caused striking dermal and epidermal changes including deep wrinkles, psoriasis, and dyskeratosis [17,19]. Endogenous photosensitizers and chromophores can be interacted with both UVA and UVB, resulting in reactive oxygen species (ROS) production which leads to damaged DNA, proteins, and lipids; however, UVB can interact directly with DNA and synthesize dipyrimidine photoproducts including pyrimidine–pyrimidone [20,21].

We demonstrated that dietary suberic acid administration in UVB-irradiated hairless mice attenuated skin dryness. The major player associated with hydration is hyaluronic acid, a non-sulfated acidic carbohydrate composed of repeating disaccharide molecules. Hyaluronic acid is produced in the membrane by hyaluronic acid synthases (HAS1, HAS2, and HAS3) [22,23]. HAS2 seems to be the main subtype responsible for hyaluronic acid synthesis, given that the HAS2 decrease extent closely relates to hyaluronic acid production downregulation [24,25,26]. Although the causes regulating hyaluronic acid synthesis decrease during photoaging and are roughly unknown, collagen degradation is considered a possible factor for hyaluronic acid loss in response to UVB. Collagen fragment degradation stimulates αvβ3-integrins and in turn inhibits Rho kinase pathway, thereby resulting in downregulated HAS2 expression [22,24].

In the present study, we found that dietary suberic acid downregulated the MAPK phosphorylation and its downstream molecules in UVB-irradiated hairless mouse skin. MAPK is related to various cellular responses to a broad array of stimuli, such as growth factors, hormones, oxidative stress, and cytokines [27,28,29,30]. Although it is believed that intracellular ROS generation is mainly responsible for MAPK activation during photoaging [31,32], we cannot exclude the possibility that dietary suberic acid attenuates the MAPK cascade in a different manner. Suberic acid is especially known as a ligand of olfactory receptor 544, which belongs to a large G-protein coupled receptor family, stimulating cAMP production in a variety of cells [33,34,35]. Recently, the cAMP pathway has also been reported to suppress ERK, JNK and p38 activities and suppress MMP expression in dermal fibroblasts [36,37]. Nevertheless, further research is needed to determine the mechanism responsible for MAPK signaling pathway downregulation in the skin of UVB-exposed mice fed a diet containing suberic acid.

The underlying mechanism for the anti-photoaging effect of suberic acid could be associated with the upregulated expression of TGF-β protein, resulting in SMAD 2/3 phosphorylation, and subsequent mRNA expression of COL1A1, COL1A2, and COL3A1 and downregulated MAPK phosphorylation, leading to AP-1 inactivation and subsequent mRNA expression of MMP1a, MMP1b, MMP3a, and MMP9. Over the past few decades, the importance of maintaining ECM homeostasis has motivated extensive research into molecular mechanisms that result in ECM synthesis and degradation. These studies have emphasized the importance of the TGF-β pathway for ECM synthesis by generally activating SMAD 2/3, while the MAPK pathway has been widely accepted as a crucial factor for ECM degradation via AP-1/MMP [5,6,7,11]. It has also been suggested that these two pathways are not completely separated and more intricately connected than previously thought. For example, TGF-β is reported to increase the tissue inhibitor of metalloproteases (TIMP-1) and plasminogen activator inhibitor 1 (PAI-1), both of which directly decrease MMP expression [38]. Furthermore, it is well known that AP-1 downregulates procollagen gene level by suppressing TGF-β/SMAD pathway [31,38]. In our experiment, the phenotypic improvement of photoaging may have potentially been accelerated through the two main synergic pathways.

## 5. Conclusions

In summary, our studies show that suberic acid supplementation contributes to skin photoaging reduction as evidenced by reduced wrinkle formation, dryness, and skin thickness in UVB exposed hairless mice. Furthermore, we also demonstrated that suberic acid treatment upregulated TGF-β signaling and downregulated the MAPK signaling pathways, which were mainly responsible for ECM synthesis and breakdown, respectively. Our results indicate the great potential for suberic acid to be further developed as an anti-photoaging agent.

## Figures and Tables

**Table 1:** Primer sequences.

Type	Gene Description	Sequences (5’→3)
Mouse	Collagen type I alpha 1 chain (*COL1A1*)	F: GGCAACAGTCGCTTCACCTA
		R: AGTCCGAATTCCTGGTCTGG
	Collagen type I alpha 2 chain (*COL1A2*)	F: CCCAGAGTGGAACAGCGATT
		R: ATGAGTTCTTCGCTGGGGTG
	Collagen type III alpha 1 chain (*COL3A1*)	F: TAACCAAGGCTGCAAGATGG
		R: ACCAGTGCTTACGTGGGACA
	Matrix metalloproteinase 1a (*MMP1a*)	F: AGTACTACAACTGACAACCCAAGA
		R: CCTGTTCCTGTTTTCAGAGCC
	Matrix metalloproteinase 1b (*MMP1b*)	F: CCTTCCCCAAATCCCATCCA
		R: CACATCGATCAAAGGTTCTGGC
	Matrix metalloproteinase 3 (*MMP3*)	F: ACTCCCTGGGACTCTACCAC
		R: GGTACCACGAGGACATCAGG
	Matrix metalloproteinase 9 (*MMP9*)	F: GTGGACCATGAGGTGAACCA
		R: ACTGCACGGTTGAAGCAAAG
	Hyaluronic acid synthase 1 (*HAS1*)	F: CTATGCTACCAAGTATACCTCG
		R: TCTCGGAAGTAAGATTTGGAC
	Hyaluronic acid synthase 2 (*HAS2*)	F: CGGTCGTCTCAAATTCATCTG
		R: ACAATGCATCTTGTTCAGCTC
	Hyaluronic acid synthase 3 (*HAS3*)	F: GATGTCCAAATCCTCAACAAG
		R: CCCACTAATACATTGCACAC
	Glyceraldehyde-3-phosphate dehydrogenase (*GAPDH*)	F: GTGATGGCATGGACTGTGGT
		R: GGAGCCAAAAGGGTCATCAT
